# Eighth Annual Report of the Pennsylvania Training School for Feeble-Minded Children

**Published:** 1861-09

**Authors:** 


### ART. XIII.—*Eighth Annual Report of the Pennsylvania Training School for Feeble-Minded Children.* Philadelphia, 1861.

WE always hail with pleasure the yearly-recurring statement of the prosperity of our benevolent institutions. The Training-School is one of the latest of that class, which has grown up into a deserving object of our fostering care and interest. When this institution for imbeciles becomes divided, according to the suggestions of the superintendent, into a school for such as are qualified, a gymnasium for physical culture, a hospital department for the sick, and a mechanical and agricultural department for the promotion of industry, with a view to the self-support of the inmates, it will become more eminently useful than ever, successful as it has already been in the purposes for which it was initiated several years ago.

We are glad to see so much stress laid upon the physical development of the children, and an effort made to abolish routine modes of accomplishing this result, by regarding each case as a problem to be studied individually, and treated according to its requirements. In the school department a word or two learned per week is considered good progress for those who have been always silent, and those further advanced are encouraged to develop all their latent powers in every possible mode that toleration, charity, and sympathy can suggest. Parents are often deceived, however, by the happy progress of apparently hopeless cases, and are disappointed that their own children, to all appearance so much more promising, should remain so stationary, even after ardent efforts to ameliorate their condition.

The present number of inmates is nearly ninety, about two-thirds of whom are males, divided as follows:—

| | | | |
|---|---|---|---|
| Epileptics | 11 | Mutes | 21 |
| Scrofulous | 50 | Semi-mutes | 20 |
| Deformed | 4 | With defective articulation | 22 |
| With chorea | 3 | With correct articulation | 25 |
| Healthy | 15 | | |

